# Anti-corrosion performance of the synergistic properties of benzenecarbonitrile and 5-bromovanillin on 1018 carbon steel in HCl environment

**DOI:** 10.1038/s41598-017-17867-0

**Published:** 2017-12-14

**Authors:** Roland Tolulope Loto

**Affiliations:** Department of Mechanical Engineering, Covenant University, Ota, Ogun State Nigeria

## Abstract

The synergistic properties of the combined admixture of benzenecarbonitrile and 5-bromovanillin (BNV) on the corrosion resistance of 1018 carbon steel in 1 M HCl was analysed with potentiodynamic polarization technique, weight loss method, micro-analytical studies and ATF-FTIR spectroscopy. Results obtained show the admixed organic compound was effective with optimal corrosion inhibition values of 99.33% and 90.34% at 1.25% BNV concentration from both electrochemical methods due to the effective inhibition action and passivation characteristics of the protonated inhibitor molecules in the acid solution. Primary amines, stretch alkyl halides and C-H triple bond functional groups of the molecules were observed to actively adsorb during the corrosion inhibition reaction from ATF-FTIR spectroscopic analysis. Calculations from thermodynamic evaluation confirmed cationic adsorption mechanism to be chemisorption obeying the Langmuir and Frumkin adsorption isotherm. Micro-analytical observations of the inhibited carbon steel morphology significantly contrast the unprotected steel due to visible surface deterioration and presence of micro/macro-pits. The organic derivatives showed mixed type inhibition reactions.

## Introduction

Carbon steel corrosion in aggressive industrial environments is of immense economic importance due to the extensive application of the alloy. They are employed as a construction material, storage containers, reservoirs and equipment parts in chemical processing plants, mining industry, power generation and petrochemical industries. In oil and gas they are applied for pipe work in down hole tubular, flow lines and transmission pipelines^1–4^. Under a variety of conditions they are used for handling alkaline, acidic and salt solutions. Solutions containing as low as trace amounts of chlorides, sulphates and nitrate ions in aqueous media are notably aggressive and accelerate corrosion of carbon steel. Acid solutions are utilized in the removal of impurities, rust and scales in mining and extraction processes such as in oil-well acidizing, industrial acid cleaning, acid-descaling and acid pickling; however in the presence of chemical compounds known as inhibitors, corrosion of carbon steel is effectively controlled. Most well-known compounds for corrosion inhibition have been recently discovered to be carcinogenic and environmentally unfriendly^5,6^. Recently in industrial plants it has become a common practice to use chemical inhibitors based environmental sustainability and cost in addition to their inhibition efficiency^7^. A number of organic chemical compounds have been known to be environmentally friendly and effective corrosion inhibitors. Generally, these compounds adsorb on the metal surface and form a passive film which suppresses the redox electrochemical processes responsible for the propagation of corrosion^8–11^. These compounds contain heteroatoms (nitrogen, oxygen, sulphur etc.) which protonates in solution, donating unshared electron pairs, unsaturated bonds (such as double bonds, or triple bonds), and plane conjugated systems including all kinds of aromatic cycles in their structures^12–14^. Synergistic effect of chemical compounds is one of the most important methods in corrosion inhibition process which serves as the basis for most of the modern corrosion inhibiting admixtures^15,16^. In furtherance of the drive for cost effective and environmentally benign corrosion inhibitors, this research focuses on the evaluation of the corrosion inhibition effect of the synergistic combination of benzenecarbonitrile and 5-bromovanillin on 1018 carbon steel, a highly applicable metallic alloy in dilute HCl solution.

## Experimental Methods

### Materials and preparation

**Figure 1:**
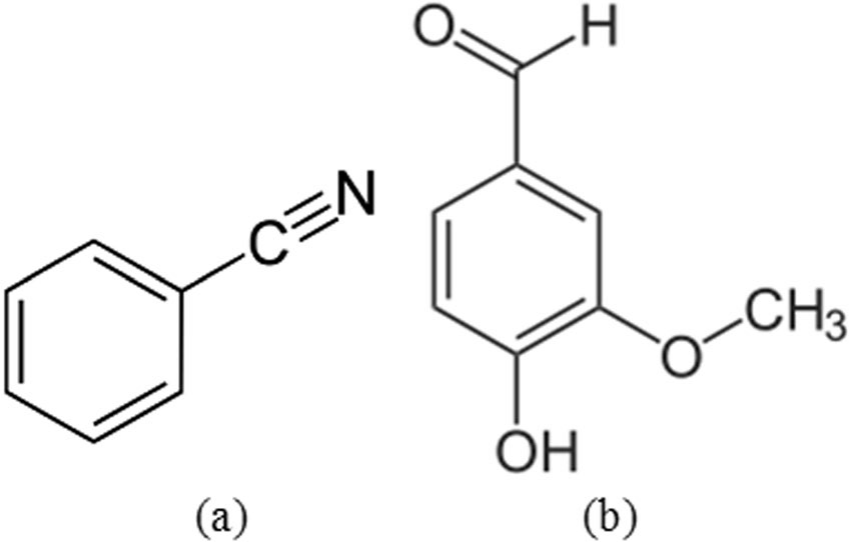
Molecular structure of (**a**) benzenecarbonitrile (**b**) 5-bromovanillin.

1018 carbon steel (1018CS) obtained from the tie rod linkage of an automobile has a basic composition (wt. %) depicted in Table 1. The cylindrical steel specimens (length, 1 cm and diameter, 1 cm) were metallographically prepared after machining with silicon carbide abrasive papers (80, 320, 600, 800 and 1000), washed with distilled water and acetone, and kept in a desiccator for weight loss and potentiodynamic polarization test according to ASTM G1 - 03(2011)^17^. Benzenecarbonitrile and 5-bromovanillin purchased from BOC Sciences, USA were the organic compounds evaluated. Benzenecarbonitrile is an aromatic, translucent organic compound with a sweet almond odour. It used mainly as an antecedent to the resin benzoguanamine. They have a chemical formula of C_7_H_5_N and molar mass of 103.12 g/mol. 5-bromovanillin is a carbolic aldehyde organic compound with the chemical formula of C_8_H_8_O_3_ and molar mass of 152.15 g/mol. It is one of the constituents of vanilla bean extract and is used as a flavouring agent in foods, beverages, and pharmaceuticals. The molecular structures benzenecarbonitrile and 5-bromovanillin are shown in Fig. 1. Their combined admixture (BNV) in equal ratios (1:1) was synthesized in molar concentrations of 9.79 × 10^−3^, 1.96 × 10^–2^, 2.94 × 10^−2^, 3.92 × 10^−2^, 4.90 × 10^−2^, 5.88 × 10^−2^, in 200 mL of 1 M HCl solution, prepared from standard grade of HCl acid (37%) with deionized water.

**Table 1:** Composition (wt. %) of 1018CS.

Element	Mn	P	S	C	Fe
**% Composition (1018CS)**	0.8	0.04	0.05	0.16	98.95

### Potentiodynamic polarization test

Polarization test was performed at 30 °C. A triple electrode workstation (platinum rod counter, electrode, silver chloride reference electrode with 3 M KCl electrolyte at a pH of 6.5 and cylindrical 1018CS working electrodes) within a glass cell containing 200 mL of HCl/BNV inhibitor formulation were connected to Digi-Ivy 2311 potentiostat to monitor the test. The working electrodes were encased in resin mounts with limited surface area of 0.79 cm^2^. Polarization curves were plotted at scan rates of 0.0015 V/s at potentials of −1.25 V to +0.5 V^18,19^. Corrosion current density *J*_cr_, (A/cm^2^) and corrosion potential, *E*_cr_ (V) values were determined by Tafel extrapolation. Corrosion current, *I*_cr_ (A) was calculated from the intercept of the cathodic and anodic polarization plots^20,21^. Corrosion rate, *C*_R_ (mm/y) was determined from the formula below;

1 $${C}_{R}=\frac{0.00327\times {J}_{{\rm{cr}}}\times {E}_{{\rm{qv}}}}{D}$$

where *E*_qv_ is the equivalent weight (g) of metal, 0.00327 is a constant^22^, and *D* is the density (g/cm^3^) Inhibition efficiency values, *η* (%) were calculated from corrosion rate results (equation 2);

2 $${\eta }_{2}=[1-(\frac{{C}_{{\rm{R2}}}}{{C}_{{\rm{R1}}}})]\times 100$$

*C*_R1_ and *C*_R2_ are the corrosion rate without and with BNV compound.

Polarization resistance, *R*_p_, (Ω) was determined from equation 3;

3 $${R}_{p}=2.303\frac{{B}_{{\rm{a}}}{B}_{{\rm{c}}}}{{B}_{{\rm{a}}}+{B}_{{\rm{c}}}}(\frac{1}{{I}_{{\rm{cr}}}})$$

Where *B*_a_ is the anodic Tafel slope (V/dec) and *B*_c_ is the cathodic Tafel slope (V/dec).

### ATF-FTIR Spectroscopy and optical microscopy characterization

HCl solutions with BNV compound at predetermined concentration, before and after the electrochemical tests were exposed to infrared ray beams with Bruker Alpha FTIR spectrometer at 375 to 7500 cm^−1^ wavelength and 0.9 cm^−1^ resolution. The resulting ATF-FTIR absorption plot consisting of spectra peaks were assessed and correlated with the theoretical ATF-FTIR absorption Table to determine the functional groups responsible for corrosion inhibition of 1018CS. Optical micrographs of BNV inhibited and non-inhibited 1018CS morphology were analysed after corrosion test with Omax trinocular using ToupCam software for analysis.

### Weight loss measurement

1018CS steel samples individual hanged within 200 mL of 1 M HCl solution for 240 h were weighed at 24 h interval^23^. Corrosion rate, *C*_R_ (mm/y) was calculated as follows^24^;

4 $${C}_{R}=[\frac{87.6\omega }{DAt}]$$

*ω* is the weight loss (g), *D* is the density (g/cm^3^), *A* is the total exposed surface area of 1018CS sample (cm^2^) and 87.6 is a constant. *t* is the time (h). Inhibition efficiency values (*η*) were calculated from the formula below;

5 $$\eta =[\frac{{\omega }_{1}-{\omega }_{2}}{{\omega }_{1}}]\times 100$$

*ω*_1_ and *ω*_2_ are the weight loss at specific BNV concentrations. Surface coverage values were calculated from equation 6 ^25,26^:

6 $$\theta =[1-\frac{{\omega }_{2}}{{{\rm{\omega }}}_{1}}]$$

where *θ* is the extent of BNV coverage on 1018CS. *ω*_**1**_ and *ω*_2_ are the weight loss of each 1018CS at predetermined BNV concentration in 1 M HCl solution.

## Result and Discussion

### Potentiodynamic polarization studies

**Figure 2:**
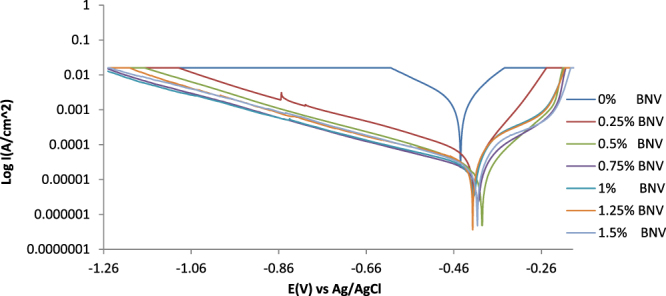
Potentiodynamic polarization plots for 1018CS in (0.25–1.5%) BNV/1 M HCl solution.

**Figure 3:**
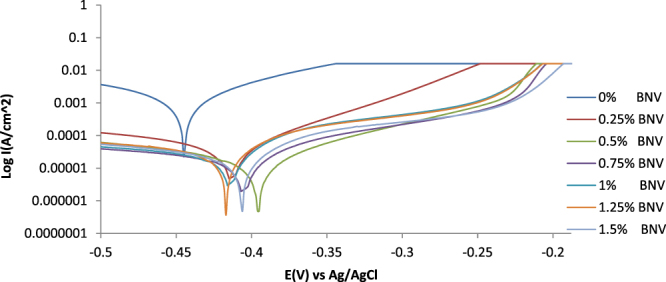
Magnified image of the potentiodynamic polarization plots for 1018CS in (0.25–1.5%) BNV/1 M HCl solution.

Results from the potentiodynamic polarization plots (Fig. 2) of the electrochemical reaction of BNV on 1018CS in 1 M HCl solution are shown in Table 2. Figure 3 is a magnified view of Fig. 2. The potentiostatic values in Table 2 show significant difference in corrosion rates and other parameters between the uninhibited (0% BNV) and BNV inhibited samples (0.25–1.5% BNV). 1018CS at 0% BNV was subjected to severe anodic dissolution from observation of the corrosion rate value at 16.93 mm/y and corrosion potential of −0.445 V. Its polarization plot shows relatively higher corrosion current density at the intercept between the anodic and cathodic polarization curves. Addition of 0.25% BNV concentration shifts the polarization plot to −0.413 V signifying slight passivation at a current density of 4.72 × 10^−5^ Acm^−2^ due to anodic inhibition, however observation of the cathodic and anodic Tafel slope values shows mixed inhibition reaction on the steel surface. The anodic Tafel slope is greater than the respective cathodic Tafel slopes due to the anodic exchange-current density value being lower than the cathodic value. Increase in concentration of BNV (0.5–1.5% BNV) caused a significant increase in passivation of the polarization plot (Fig. 3). The corrosion rate reduced further (0.25% BNV–0.5% BNV), but after 0.5% BNV further increase did not impact noticeably on the corrosion rate values. This shows BNV concentration after 0.5% has a minor effect on the corrosion rates values of 1018CS. The observation is further confirmed from the inhibition efficiency values which were generally similar.

**Table 2:** Polarization results for 1018CS in (0.25–1.5%) BNV/1 M HCl solution.

Sample	BNV Conc. (%)	BNV Conc. (M)	Corrosion Rate, *C*_R_ (mm/y)	BNV Inhibition Efficiency, *η*_2_ (%)	Corrosion Current, *I*_cr_, (A)	Corrosion Current Density, *J*_cr_ (A/cm^2^)	Corrosion Potential, *E*_cr_ (V)	Polarization Resistance, R_p_ (Ω)	Cathodic Tafel Slope, B_c_ (V/dec)	Anodic Tafel Slope, B_a_ (V/dec)
A	0	0	16.93	0	1.15E–03	1.46E–03	−0.445	34.76	−6.344	9.983
B	0.25	9.79E–03	0.55	97.95	3.73E–05	4.72E–05	−0.413	426.99	−4.689	14.580
C	0.50	1.96E–02	0.21	99.22	1.43E–05	1.81E–05	−0.395	1104.42	−4.229	11.070
D	0.75	2.94E–02	0.19	99.28	1.31E–05	1.66E–05	−0.405	1154.40	−3.538	7.644
E	1.00	3.92E–02	0.19	99.29	1.29E–05	1.64E–05	−0.414	1227.64	−3.391	6.674
F	1.25	4.90E–02	0.18	99.33	1.23E–05	1.55E–05	−0.417	1318.35	−3.330	6.331
G	1.50	5.88E–02	0.19	99.30	1.27E–05	1.61E–05	−0.406	1303.17	−3.201	5.772

The visible decrease in potential of the polarization plots after 0.25% BNV, is due to surface coverage by BNV molecules whereby it stifles the redox electrochemical mechanisms responsible for corrosion^27,28^. This observation is clearly visible on the polarization plots wherewith after 0.25% BNV passivation is visible on the anodic portion of the polarization plot. The passivation signifies extended delay in the polarization of 1018CS and increases in its polarization resistance before failure under applied potential. This phenomenon is associated with stainless steels that undergo localized corrosion due to the passive film on the steel’s surface^29–31^. During upward scanning the steel eventually corrodes, but the plots shows that the steel pitted resulting from localized corrosion. This summation is confirmed from optical microscopy analysis where the presence of microscopy pits are visible on the inhibited steel sample. The polarization resistance increased with decrease in corrosion rates. The highest variation in corrosion potential with respect to the value at 0% BNV is 55 mV in the anodic direction, thus BNV is a mixed type inhibitor^32,33^.

BNV being an organic compound adsorbs on metallic surfaces after protonating in the acid solution. Adsorption depends on the physicochemical properties of the inhibitor molecules related to its functional groups, possible steric effects, electronic density of donor atoms and presence of heteroatoms. The possible interaction of p-orbitals of BNV with d-orbitals of 1018CS surface atoms resulting from electrostatic attraction due to preadsorbed Cl^−^ ions induces strong adsorption leading to the formation of a corrosion protecting film^34^. Transfer of lone pairs of electrons on the N and O atoms to 1018CS surface to form coordinate type linkage is favoured by the presence of vacant orbital in the iron atom of 1018CS^35^. In the acid solution 1018CS acts as an electrophile, whereas the nucleophile centers of BNV molecule with free electron pairs readily available for sharing, results in bond formation and the formation of an impenetrable protective barrier^36,37^. This influenced the kinetics of 1018CS dissolution as the protective inhibitor film separates the steel from the corrosive anions in the acid solution.

### ATF-FTIR spectroscopy analysis

Benzenecarbonitrile component of BNV being an aromatic organic compound consists of cyclic and planar molecules with a ring of resonance bonds. Its basic functional group is the cyano group (−C≡N, containing trivalent nitrogen) which is attached to one carbon atom with the general formula RC≡N. They are polar with high dipole moments which creates strong electrostatic attraction with the 1018CS surface. 5-bromovanillin component of BNV is a phenolic aldehyde with the aldehyde, hydroxyl, and ether functional group. Considering the structure of BNV different points where it can interact with 1018CS can be established. The free electron pairs on N and O, and the π-electrons from the aromatic rings are capable of forming covalent bonds with Fe substrate metal of 1018CS. The N atom of the amino group attached to the aromatic ring enhances the BNV molecule to strongly adsorb onto the steel^38,39^.

**Figure 4:**
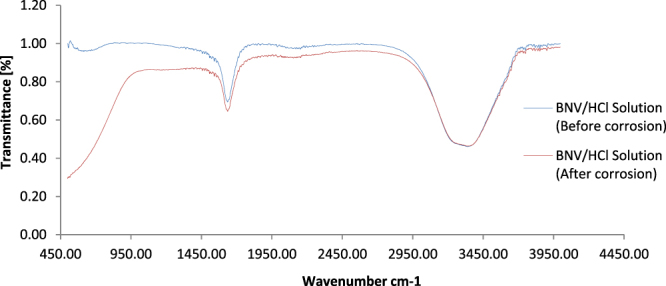
ATF-FTIR spectra of BNV/1 M HCl solution before and after 1018CS corrosion test.

Designation of the functional groups of atoms or bonds within molecules involved in BNV (combined admixture) inhibition on 1018CS after hydrolysis in HCl, was done through IR spectroscopy and correlated with the theoretical IR Table^40,41^ for identification. The IR spectra of 1 M HCl/BNV solutions before and after the corrosion test are shown in Fig. 4. The spectra diagrams show the same peak configuration for both solutions between wavelengths of 3067.46 cm^−1^ and 3573.48 cm^−1^ consisting of alkenes, aromatics, alkynes (terminal), carboxylic acids, primary and secondary amines, amides, alcohols and phenols functional groups (=C–H stretch, C–H stretch, –C(triple bond)C–H: C–H stretch, O–H stretch, N–H stretch, O–H stretch and H–bonded bonds) within BNV compound due to limited or negligible adsorption. At wavelengths between 1592.27 cm^−1^ and 3067.46 cm^−1^, slight decrease in transmittance intensities of the spectra peak was observed due to adsorption of aromatics, primary amines, alkenes, alpha, beta–unsaturated aldehydes and ketones, saturated aliphatic, alpha, beta–unsaturated esters, aldehydes, esters, carboxylic acids, carbonyls (general), nitriles, alkynes, alkanes, alkenes, aromatics, alkynes (terminal), primary, secondary amines, amides, alcohols and phenols functional groups present. These consists of C=O stretch, –C=C– stretch, N–H bend, C–H stretch, H–C=O: C–H stretch, C(triple bond)N stretch, C(triple bond)C stretch and C–C stretch (in–ring) bonds.

Significant variation in transmittance signifying strong molecular interaction and adsorption was observed between wavelengths of 526.23 cm^−1^ and 1592.27 cm^−1^. The functional groups at these wavelengths consists of most of the earlier mentioned functional groups, in addition to nitro compounds, aromatic amines, ethers, alkyl halides, aliphatic amines, alkenes, carboxylic acids, primary, secondary amines. Previous research has shown that compounds with amines, carboxylic acids and alcohols functional groups are effective corrosion inhibitors^42,43^. The C–Cl (stretch alkyl halides) resulting from replacement one of the H atom of alkyne molecule with Cl atom is due to the electrostatic attraction between the protonated BNV molecules and Cl^−^ anions on the steel surface. BNV replaces the Cl^−^ ions due to competitive adsorption onto the steel surface.

### Weight loss measurements

**Figure 5:**
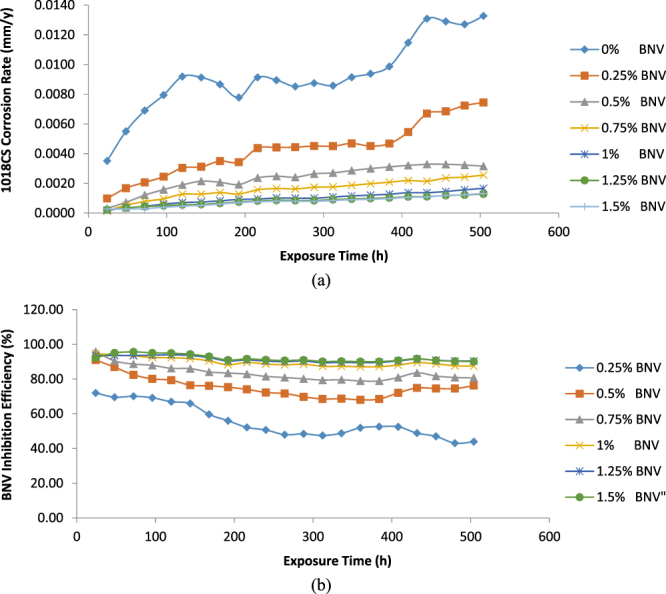
Plot of (a) 1018CS corrosion rate versus exposure time (b) BNV inhibition efficiency versus exposure time in 1 M HCl at 0–1.5% BNV concentration.

Results obtained from weight loss measurement for 1018CS weight loss (*ῶ*) and corrosion rate (*C*_R_), and BNV percentage inhibition efficiency (ɲ) in HCl acid solution at 504 h are depicted in Table 3. Figure 5(a,b) depict the graphical illustration of 1018CS corrosion rate and BNV percentage inhibition efficiency versus exposure time in the acid solution. The corrosion rates of the carbon steel sample at 0% BNV (13.270 mm/y, 504 h) differs from the values obtained for 0.25–1.5% BNV concentrations. In the absence of BNV inhibitor, 1018CS undergoes severe accelerated corrosion, the rate of which continued to increase with increase in exposure time till 504 h due to the debilitating effect of Cl^−^ ions in the acid solution. Dilute HCl acid completely ionizes in H_2_O releasing one proton (equation 7) to form Cl^−^ and H_3_O^+^ (hydronium ion). The Cl^−^ ion strongly reacts with the steel surface (equation 8) to form iron (II) chloride.

7 $${\rm{2HCl}}+{{\rm{2H}}}_{2}{\rm{O}}\to {{\rm{2H}}}_{{\rm{3}}}{{\rm{O}}}^{+}+{{\rm{2Cl}}}^{-}$$

8 $${\rm{Fe}}({\rm{s}})+2{{\rm{H}}}_{3}{{\rm{O}}}^{+}+2{{\rm{Cl}}}^{-}\to +{{\rm{FeCl}}}_{2}+{{\rm{H}}}_{2}+{{\rm{2H}}}_{{\rm{2}}}{\rm{O}}$$

**Table 3:** Result for 1018CS in 1 M HCl/0–1.5% BNV at 504 h from weight loss measurement.

Samples	Weight Loss (g)	BNV Concentration (%)	BNV Concentration (Molarity)	Corrosion Rate (mm/y)	BNV Inhibition Efficiency (%)
A	3.428	0	0	13.270	0
B	1.923	0.25	9.79E–06	7.444	43.91
C	0.817	0.5	1.96E–05	3.161	76.18
D	0.661	0.75	2.94E–05	2.557	80.73
E	0.430	1	3.92E–05	1.664	87.46
F	0.331	1.25	4.90E–05	1.282	90.34
G	0.341	1.5	5.88E–05	1.319	90.06

Addition of 0.25% BNV concentration drastically reduced the corrosion of 1018CS to 7.444 mm/y (43.91% BNV inhibition efficiency) at 504 h; however the rate of corrosion is still relatively high due to the presence of insufficient BNV molecules to counteract the action of Cl^−^ ions. At 0.5% BNV till 1.25% BNV concentration, the corrosion rate significantly reduced to negligible values (3.161–1.282 mm/y, 504 h) proportional to BNV inhibition efficiency of 76.18–90.34%. The presence of BNV stifled the redox reactions responsible for surface deterioration. This inhibits the release of oxidized Fe^2+^ resulting from the electrochemical action of Cl^−^ ions, into the acid solution. In the acid media, BNV protonates strongly with respect to concentration allowing more of its molecules to donate unshared electrons, hence increasing the functionality of the inhibitor and enabling strong adsorption onto the steel surface. Increase in BNV concentration to 1.5% at 90.06% BNV inhibition efficiency had no further influence on the corrosion rate values signifying that the optimum BNV concentration for effective corrosion inhibition is 1.25% BNV in 1 M HCl.

### Adsorption Isotherm

**Figure 6:**
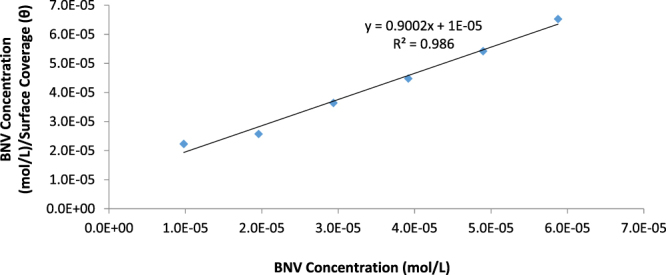
Plot of $\frac{C}{\theta }$ versus BNV concentration in 1 M HCl.

**Figure 7:**
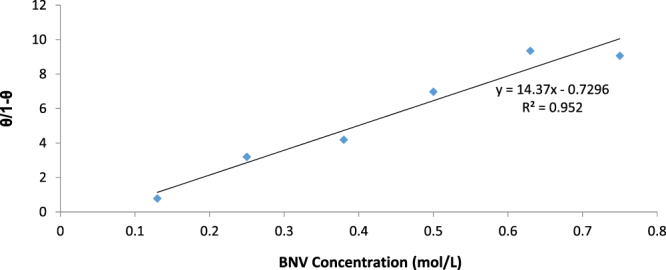
Plot of $\frac{\theta }{1-\theta }$ versus BNV concentration in 1 M HCl.

The corrosion inhibition of BNV on 1018CS is an electrochemical process through which BNV protonated BNV molecules diffuse to the metal/solution interface and attach itself onto the steel through intermolecular or electrostatic/covalent interaction. The ionization potential, properties of metal surface, electronic behaviour and the degree of ionic adsorption are the major factors that influence the mechanism and type of adsorption^44–47^. The adsorption mechanism of BNV inhibitor was determined and studied to achieve a further in-depth understanding of the molecular interaction between the compound and 1018CS. BNV adsorption strongly aligns with the Langmuir and Frumkin isotherm models as shown in Figs 6 and 7 according to the following equations.

9 $$\theta =[\frac{{K}_{{\rm{ads}}}{C}_{{\rm{BNV}}}}{1+{K}_{ads}{C}_{{\rm{BNV}}}}]$$

10 $$\mathrm{Log}[{C}_{{\rm{BNV}}}\ast (\theta /1-\theta )]=2.303\,\mathrm{log}\,{K}_{{\rm{ads}}}+2\alpha \theta $$

*θ* is the extent of BNV molecular coverage on 1018CS, *C*_BNV_ is BNV concentration in HCl solution, and *K*_ads_ is the equilibrium constant of the adsorption mechanism. The graphical illustration of $\frac{{C}_{{\rm{BNV}}}}{\theta }$ versus *C*_BNV_ showed linearity in agreement with Langmuir adsorption isotherm (Fig. 6) with a correlation coefficient of 0.986 while the graphical illustration of the Frumkin isotherm (Fig. 7) had a correlation coefficient of 0.952. The Langmuir isotherm suggests that (i) The intermolecular reaction on the metal surface is constant, (ii) The value Gibbs free energy is independent of the degree of molecular coverage and (iii) There is no lateral interaction effect resulting from the molecular reaction of adsorbates on the value of Gibbs free energy^48^. Frumkin isotherm suggests total molecular coverage at high BNV concentrations for a heterogeneous alloy and the effect of lateral interaction is significant. As a result the reactive site on the metal surface involved in adsorption interactions is taken into account.

### Thermodynamics of the corrosion process

The thermodynamics of the inhibition mechanism discusses the adsorption strength of BNV compound on 1018CS. The adsorption strength is proportional to the extent of H_2_O molecules (n) dislodge by BNV. Data of Gibbs free energy (${\rm{\Delta }}{G}_{ads}^{0}$) for the molecular interaction is shown in Table 4 was evaluated from equation 11^49^.

11 $${\rm{\Delta }}{G}_{{\rm{ads}}}=-2.303RT\,\mathrm{log}[55.5{K}_{{\rm{ads}}}]$$

55.5 is a constant for molar concentration of water in the solution, *R* is the universal gas constant, *T* is the absolute temperature and *K*_ads_ is the equilibrium constant of adsorption. *K*_ads_ is related to surface coverage ($\theta$) from the Langmuir equation (equation 9).

**Table 4:** Result for Gibbs free energy (${\rm{\Delta }}{G}_{ads}^{0}$), surface coverage (θ) and equilibrium constant of adsorption (*K*_ads_) for BNV adsorption on 1018CS.

Samples	BNV Concentration (M)	Surface Coverage (θ)	Equilibrium Constant of adsorption (*K*_ads_)	Gibbs Free Energy, ∆*G*_ads_ (Kjmol^−1^)
A	0	0	0	0
B	9.794E-06	0.439	79928.8	−37.93
C	1.959E-05	0.762	163284.0	−39.70
D	2.938E-05	0.807	142599.8	−39.36
E	3.917E-05	0.875	178041.8	−39.91
F	4.897E-05	0.903	190965.4	−40.08
G	5.876E-05	0.901	154225.9	−39.56

Flaws, discontinuities and impurities on stainless steels significantly influence ${\rm{\Delta }}{G}_{ads}^{0}$ values as the surface coverage value of BNV changes^50^. Organic molecular adsorption being a substitutional reaction involving the removal of H_2_O molecules from metal surface through hydrophobic part of functional groups is proportional to the degree of metal cations diffused into the acid solution. The amount of cations released to the acid solution is proportional to the degree of BNV coverage due to changes in interaction energy with water molecules as BNV cations adsorb on the steel^51^. The negative values of ${\rm{\Delta }}{G}_{ads}^{0}$ show the spontaneous characteristics of the adsorption mechanism and continuity of the molecular film layer on the metal surface. The highest ${\rm{\Delta }}{G}_{ads}^{0}$ value obtained is −40.08 KJmol^−1^ at 1.25% BNV while the lowest value is −37.93 KJmol^−1^ at 0.25% BNV. This observation is consistent with chemisorption adsorption mechanism^52,53^. After 1.25% BNV the ${\rm{\Delta }}{G}_{ads}^{0}$ value slightly decreased, confirming the observation from weight loss measurement that the optimal BNV concentration for effective corrosion inhibition of 1018CS is 1.25%.

### Optical Microscopy analysis

**Figure 8:**
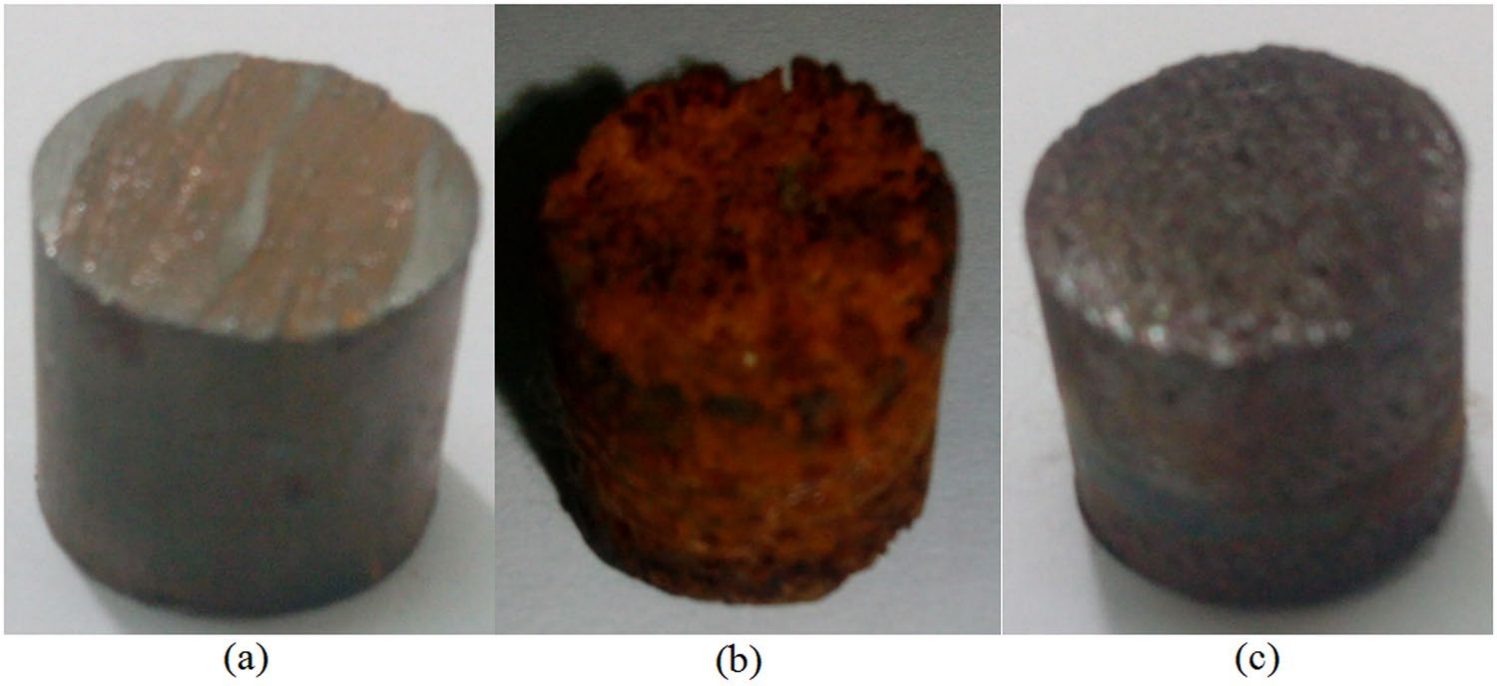
Macro-images of 1018CS samples (**a**) before corrosion test, (**b**) after corrosion without BNV and (**c**) after corrosion with BNV.

**Figure 9:**
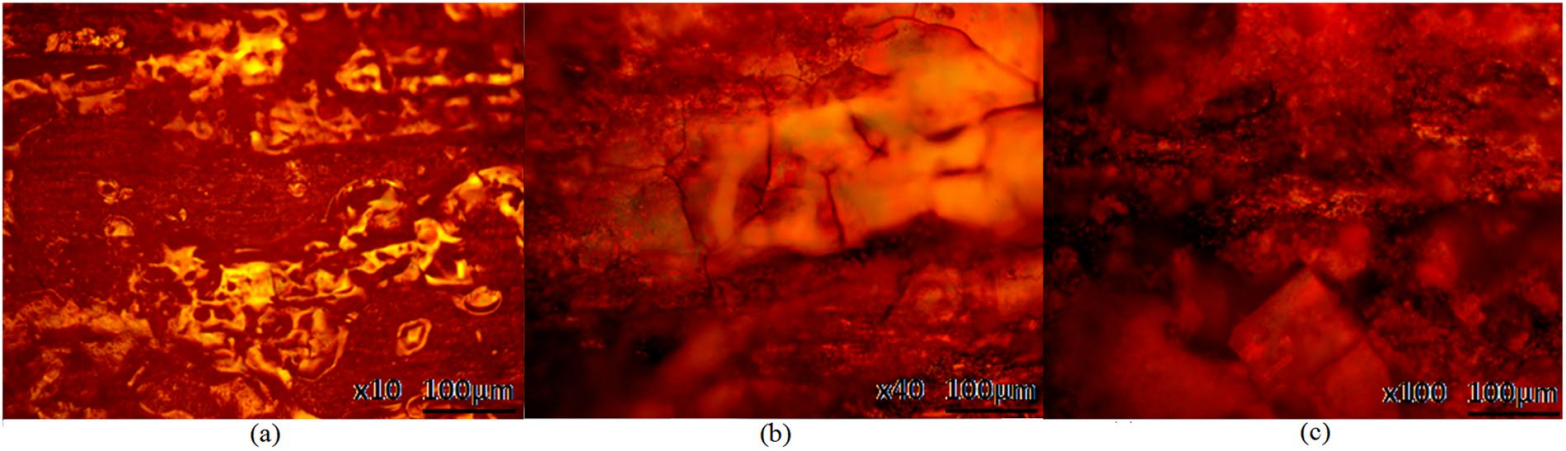
Micrographs of 1018CS samples before corrosion test (**a**) mag. x10, (**b**) mag. x40 and (**c**) mag. x100.

**Figure 10:**
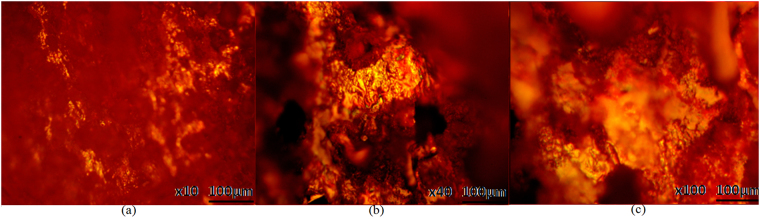
Micrographs of corroded 1018CS samples after corrosion without BNV (**a**) mag. x10, (**b**) mag. x40 and (**c**) mag. x100.

**Figure 11:**
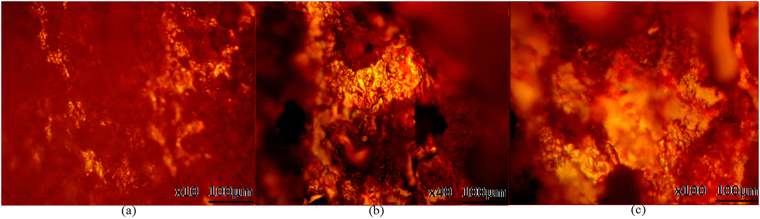
Micrographs of inhibited 1018CS samples after corrosion with BNV (**a**) mag. x10, (**b**) mag. x40 and (**c**) mag. x100.

The macro-images of 1018CS samples before corrosion, and after corrosion in and without the presence of BNV compound are shown from Fig. 8(a–c). Figures 9(a–c), 10(a–c) and 11(a–c) shows the micro-analytical image of 1018CS samples before corrosion, after corrosion without BNV and after corrosion with BNV compound at mag. x10, x40 and x100 respectively. The corroded 1018CS sample in Figs 8(b) and 10(a–c) without BNV addition confirms the result from coupon measurement and potentiodynamic polarization. The Cl^−^ ions in the acid solution severely degraded the total surface and morphology of 1018CS during the exposure hours. General corrosion best explains the topographic damage on the steel as corrosion has significantly eaten inwards on the steel. The steel contrast the images of the steel sample before corrosion test [Figs 8(a) and 9(a–c)] as it best explains the reason why carbon steel components, parts and materials fail in chloride environments. Studying 1018CS samples after corrosion in the presence of BNV (Figs 8(c) and 11(a–c), the inhibition effect of BNV is clearly visible as the steel retains its viability. BNV compound extended the lifespan and service life of 1018CS through formation of a passive, adherent covering over the entire surface of the steel. However limited microscopic pits can be observed, this could be as a result of breakages in the protective film. The inhibition effect of BNV is through adsorption as earlier discussed whereby inhibitor block the reactive sites of the steel surface inhibiting the anodic reaction and in effect the redox electrochemical process.

## Conclusion

BNV effectively improved the corrosion resistance of 1018 carbon steel in dilute HCl acid solution. The admixed organic compound adsorbed strongly onto the carbon steel, passivating it through electrostatic attraction and covalent bonding, resulting from chemisorption mechanism onto the steel surface. The inhibition efficiency values remained sufficiently high from the onset of the exposure hours till the end due to the inhibition reaction of the molecular functional groups and heteroatoms of the compounds which strongly altered the mechanism of the electrochemical process and protecting the steel from corrosion. The inhibition property of the compound was determined to be anodic type inhibitor.
